# Mechanosensitive Stem-Cell Genes and Klotho in Atherosclerotic Aortas: Regulating Spatially Deranged Expression Patterns Using Colchicine Regimens

**DOI:** 10.3390/jcm11216465

**Published:** 2022-10-31

**Authors:** Konstantinos S. Mylonas, Panagiotis Sarantis, Alkistis Kapelouzou, Michalis V. Karamouzis, Emmanouil I. Kapetanakis, Konstantinos Kontzoglou, Dimitrios C. Iliopoulos, Nikolaos Nikiteas, Dimitrios Schizas

**Affiliations:** 1 Department of Cardiac Surgery, Onassis Cardiac Surgery Center, 17674 Athens, Greece; 2 Laboratory of Experimental Surgery and Surgical Research “N.S. Christeas”, School of Medicine, National and Kapodistrian University of Athens, 15772 Athens, Greece; 3 Department of Biological Chemistry, School of Medicine, National and Kapodistrian University of Athens, 11527 Athens, Greece; 4 Center of Clinical, Experimental Surgery & Translational Research, Biomedical Research Foundation Academy of Athens, 11527 Athens, Greece; 5 Third Department of Surgery, School of Medicine, National and Kapodistrian University of Athens, Attikon University Hospital, 12462 Athens, Greece; 6 Fourth Department of Cardiac Surgery, Hygeia Hospital, 15123 Athens, Greece; 7 Second Propaedeutic Department of Surgery, Laiko General Hospital, School of Medicine, National and Kapodistrian University of Athens, 11527 Athens, Greece; 8 First Department of Surgery, Laiko General Hospital, School of Medicine, National and Kapodistrian University of Athens, 11527 Athens, Greece

**Keywords:** *Klotho*, *HOXA5*, *NOTCH1*, *HIF1a*, *NANOG*, atheromatosis

## Abstract

Aims: Inflammatory dysregulation of mechanosensitive developmental genes may be central to atherogenesis. In the present seven-week model, we utilized colchicine regimens to curtail aortic atherogenesis in New Zealand White rabbits. We also explored the effect of colchicine regimens on atheroprotective (*Klotho*, *HOXA5*, *NOTCH1*, and *OCT4*) and proatherogenic (*HIF1a*, *SOX2*, *BMP4*, and *NANOG*) genes. Methods: The control (n = 6) and group A (n = 6) received standard and cholesterol-enriched chow, respectively. Groups B (n = 8) and C (n = 8) were fed hypercholesterolemic diet and were treated with colchicine plus fenofibrate or N-acetylcysteine (NAC), respectively. Results: Group A developed significantly greater thoracic and abdominal aortic atherosclerosis compared to groups B (*p* < 0.001) and C (*p* < 0.001). Combining colchicine with NAC resulted in stronger atheroprotection both in the thoracic and the abdominal aorta. In group A thoracic aortas, *Klotho* was downregulated compared to controls (95% CI: 1.82–15.76). Both colchicine regimens upregulated *Klotho* back to baseline levels (*p* < 0.001). Colchicine/fenofibrate also significantly upregulated thoracic *NOTCH1* compared to controls (95% CI: −8.09 to −0.48). Colchicine/NAC significantly reduced thoracic *NANOG* expression compared to hyperlipidemic diet alone (95% CI: 0.37–8.29). In the abdominal aorta, hypercholesterolemic diet resulted in significant downregulation of *HOXA5* (95% CI: 0.03–2.74) which was reversed with colchicine/NAC back to baseline (95% CI: −1.19 to 1.51). Colchicine/fenofibrate downregulated *HIF1a* compared to baseline (95% CI: 0.83–6.44). No significant differences were noted in terms of *BMP4*, *SOX2*, and *OCT4*. Conclusions: Overall, the aortic expression pattern of mechanosensitive genes seems to be spatially influenced by a hyperlipidemic diet and can be modified using colchicine-based therapy.

## 1. Introduction

Landmark studies have affirmed the instrumental role that inflammation plays in atherogenesis [1,2]. Several attempts have been made to utilize anti-inflammatory agents and immunomodulators as tools against atherosclerosis [3]. The seminal LoDoCo (Low Dose Colchicine trial) and COLCOT (Colchicine Cardiovascular Outcomes Trial) trials found that low-dose colchicine significantly reduces the risk of cardiovascular death not only in the setting of stable coronary artery disease (CAD) but also in acute coronary syndromes [4,5]; indeed, colchicine suppresses the activation of inflammasome NLRP3 in neutrophils and macrophages by inhibiting tubulin polymerization and microtubule generation. As a result, the production of interleukins (IL1b and IL18) is attenuated [6,7].

Fibrates also bear atheroprotective properties beyond their conventional hypolipidemic action [8,9]. As peroxisome proliferator-activated receptor-α agonists, fibrates block nuclear factor kB (NF-kB), thereby halting the expression of adhesion molecules, matrix metalloproteinases, and tissue factor [10]. Fenofibrate also inhibits the production of IL6, IL-1β, and cyclooxygenase-2 [8,11].

Additional preclinical data have shown that N-acetylcysteine (NAC) attenuates proatherogenic inflammation [12]; indeed, NAC scavenges reactive oxygen species (ROS) and other free radicals that trigger and propagate inflammatory stimuli. NAC also induces structural changes in the tumor necrosis factor-α (TNF-α) receptor and inhibits the activation of NF-κB. Furthermore, NAC hinders adipose tissue differentiation by downregulating mitogen-activated protein kinases [12]. Lastly, NAC reduces LDL (low density lipoprotein) oxidization and diminishes foam cells [13].

A growing body of literature has shown that dysregulation of Klotho and various stem-cell genes can occur due to shear stress [14,15,16,17,18,19]. This genetic derangement appears to orchestrate the development and progression of atherosclerosis [1]. Pilot work from our group confirmed that colchicine-based treatment can curtail de novo atherogenesis in the thoracic aortas of hyperlipidemic rabbits [20]. In the present study, we expanded upon that preliminary experience by assessing the impact of colchicine-based therapy on the aortic expression pattern of cardinal atheroprotective and proatherogenic mechanosensitive genes.

## 2. Materials and Methods

### 2.1. Animal Model and Experimental Design

The design of our experimental animal model has been previously described [20]. In brief, 28, eight-week-old, male, white New Zealand rabbits (*Oryctolagus cuniculus*) were procured from a farm that breeds rabbits for experimental purposes. A 1:1 ratio of study animals to cages was respected. A 12 h light/dark cycle (5:30 am to 5:30 pm) was enforced and comfortable conditions were maintained (19 ± 2 °C with 60 ± 5% relative humidity and 15 air changes/hour). Free access to food and water was allowed. Daily water consumption was measured for each rabbit individually. All study subjects were treated in line with current European Council Directives (276/33/20.10.2010). The study protocol was approved by the Veterinary Directorate of the Prefecture of Athens (approval no.: 3231/26.06.2018). Institutional Board of Review approval was also obtained by the Ethics Committee of the School of Medicine of the National and Kapodistrian University of Athens. All methods were performed in accordance with the relevant ARRIVE guidelines and regulations.

**Figure 1:**
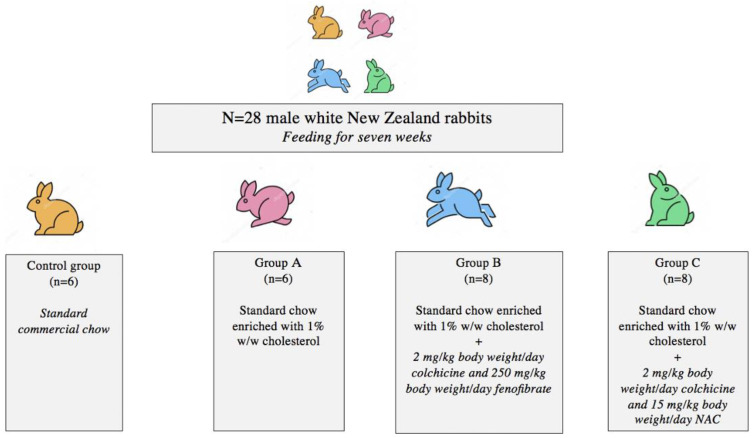
Study groups.

Following a two-week acclimatization period, study subjects were divided into four experimental groups (Figure 1). The control group (n = 6) was fed standard commercial rabbit chow (Conigli Svezzamento, S.I.V.A.M. Società Italiana Veterinaria Agricola Milano S.P.A., Casalpusterlengo (LO), Italy) comprising 37% carbohydrates, 16% protein, 4% fat, 15% fiber, 11% water, and 8% ash. Group A (n = 6) was fed a standard diet enriched with 1% w/w cholesterol. To prepare the hyperlipidemic feeds, the cholesterol was dissolved in diethyl ether; subsequently, the mixture was added to the rabbit chow. Following ether evaporation, the hyperlipidemic rations were stored at −20 °C until use.

Group B (n = 8) was fed the same hypercholesterolemic diet enriched with 2 mg/kg body weight/day colchicine and 250 mg/kg body weight/day fenofibrate. Lastly, Group C (n = 8) received the same hypercholesterolemic diet plus 2 mg/kg body weight/day colchicine and 15 mg/kg body weight/day NAC. Colchicine, fenofibrate, and NAC were pulverized and, subsequently, dissolved in tap water. Pharmaceuticals were only administered per os to avoid injection-induced stress reactions which could potentially affect the expression of developmental genes [12,21,22,23,24].

### 2.2. Tissue Preparation

Benchmark animal models have shown that four to twelve weeks of hyperlipidemic feeding are adequate for the formation of atherosclerotic lesions [22,25]; therefore, after seven weeks, all study animals were sedated with intramuscular ketamine–xylazine and euthanized via intravenous administration of sodium pentobarbital (120 mg/kg) [25]. Aortas were surgically excised from the arch down to the iliac bifurcation. Perivascular adipo-connective tissue was dissected off the vessels. Thoracic aortas were sampled between the origin of the brachiocephalic vessels and the third/fourth intercostal arteries. Abdominal aortas were sampled between the diaphragm and the iliac bifurcation. Tissue specimens were maintained in a 10% neutral buffered formalin solution for 24 h and afterwards were embedded in paraffin blocks maintaining native vertical orientation.

### 2.3. Histology

For each rabbit, 10 serial paraffin slices of 5 μm thickness were cut along the thoracic and abdominal aortic samples at equal (50 µm) intervals over a distance of 500 µm. Hematoxylin and eosin (H&E) staining was performed for quantitative morphometric analysis; additionally, the thoracic aorta, as well as the abdominal aorta, was sectioned in predefined positions, followed by the H&E stain and morphometric analysis. Histologic examination was conducted by an expert who was blind to the intervention groups. Lastly, 10 serial cryosections segments of 10μm thickness were stained with Oil Red O for the determination of the intra-plaque lipid accumulation (Figure S1).

### 2.4. Morphometric Analysis

A previously validated institutional protocol was used to perform morphometric analysis [26]. The specimens were examined under a Leica DMLS2 light microscope (Leica Microsystems Wetzlar GmbH, Germany). Digital images were acquired using a Leica DFC500 digital color camera (working resolution: 4080 × 3072 pixels) and the Leica LAS 3.6 software. Morphometric analysis of thoracic and abdominal aortic segments was performed for the quantification of atherosclerotic plaques in ImagePro Plus version 5.0 (Media Cybernetics, Bethesda, MD, USA).

### 2.5. Genes of Interest

The expression patterns of the following atheroprotective mechanosensitive genes were assessed: *α-Klotho, HOXA5, NOTCH1, OCT4* (Octamer-binding transcription factor 4). We also evaluated the expression of key proatherogenic genes, including: *NANOG*, *HIF1a* (hypoxia-inducible factor 1a), *SOX2* (Sry-related HMG box 2), and *BMP4* (bone morphogenetic protein 4).

### 2.6. RNA Extraction—cDNA Synthesis

For each animal, all-layer samples of thoracic and abdominal aortic tissue specimens were placed in RNAlater^®^ solution (Ambion, Austin, TX, USA) and stored at 4 °C for subsequent RNA extraction. Given that the atherosclerotic lesions were diffusely spread throughout the aortas, samples were randomly selected. In total, 100–200 mg of tissue was utilized from each experimental animal (50–100 mg thoracic aorta and 50–100 mg abdominal aorta). Total RNA was extracted using the RNeasy Mini Kit (Qiagen, Hilden, Germany), according to the manufacturer’s instructions [27]. RNA was quantified using a Thermo Scientific NanoDrop™ Lite Spectrophotometer (Thermo Scientific, Waltham, MA, USA). RNA quality was assessed by the A_260_/A_280_ ratio. Complementary DNA (cDNA) was produced when the A_260_/A_280_ ratio was greater than 1.8 [27]. Complementary DNA was synthesized using the PrimeScript RT reagent kit (Takara Bio, Kusatsu, Shiga, Japan), according to the manufacturer’s protocol.

### 2.7. Quantitative Real-Time Polymerase Chain Reaction (qRT-PCR)

The qPCR reaction was performed using cDNA along with KAPA SYBR^®^ FAST qPCR Master Mix (2X) Kit (KK4602, Sigma-Aldrich, St.Louis, MO, USA), forward/reverse primers, and water. All genes of interest for all experimental animals were analyzed at the same time along with a control gene that is universally expressed in cells (glyceraldehyde 3-phosphate dehydrogenase—GAPDH) (housekeeping gene). Amplification of cDNA (C_T_) values were estimated for all reactions. The level of target mRNA was estimated by relative quantification and normalized to GAPDH expression. Table S1 summarizes the primer sequences that were utilized.

### 2.8. Statistical Analysis

One-way analysis of variance (ANOVA) was performed with Tukey’s correction to account for multiple comparisons. Mean differences (MD) with 95% confidence intervals (95% CI) were calculated (in μm). A *p*-value < 0.05 was considered statistically significant. All *p*-values were two-sided. All statistical calculations were performed using Stata/BE 17.0 for Mac (StataCorp, 4905 Lakeway Drive, College Station, TX, USA) and GraphPad Prism version 4.03 (GraphPad Inc, San Diego, USA).

## 3. Results

### 3.1. Atherosclerotic Burden in Thoracic and Abdominal Aortic Specimens

Controls showed no atheromatosis in their thoracic and abdominal aortas. Group A animals had significantly more extensive thoracic atherosclerotic lesions compared to animals that received colchicine combined with fenofibrate (MD: 13.7, 95% CI: 7.5 to 19.8, *p* < 0.001) and NAC (MD: 20.3, 95% CI: 14.1 to 26.5, *p* < 0.001). Similarly, group A developed significantly more severe abdominal aortic atherosclerosis compared to groups B (MD: 12.6, 95% CI: 7.1 to 18.2, *p* < 0.001) and C (MD: 23.8, 95% CI: 18.2 to 29.3, *p* < 0.001). Combining colchicine with NAC instead of fibrate resulted in a significantly greater reduction in the extent of thoracic (MD: 6.6, 95% CI: 0.9–12.3) and abdominal aortic atheromatosis (MD: 11.1, 95% CI: 6.0–16.3).

### 3.2. α-Klotho Expression

#### 3.2.1. *α-Klotho* in Thoracic Aortic Specimens

**Figure 2:**
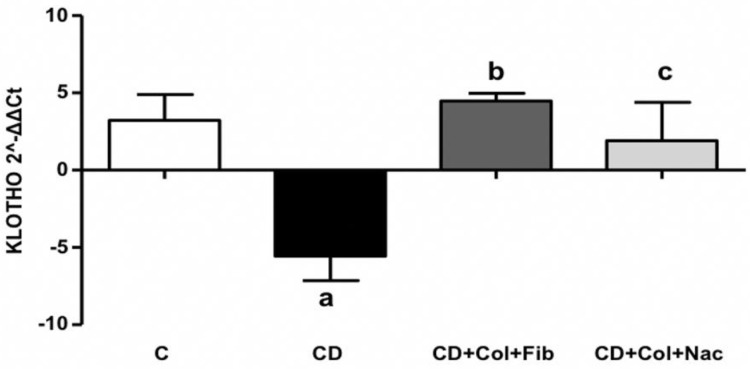
Comparison of *α-Klotho* expression in rabbit thoracic aortas. C: control, CD: cholesterol diet (group A), Col: colchicine, Fib: fibrate, NAC: N-acetylcysteine. Letters mark statistical significance between groups as follows: a: C vs. CD; b: C vs CD+Col+Fib; c: C vs. CD+Col+Nac.

Thoracic aortic *α-Klotho* expression was significantly reduced in the context of unmedicated hyperlipidemia (MD: 8.79, 95% CI: 1.82 to 15.76, *p* < 0.001). Both colchicine/fenofibrate (MD: −10.04, 95% CI: −17.00 to 3.07, *p* < 0.001) and colchicine/NAC (MD: −7.47, 95% CI: −13.83 to −1.11, *p* < 0.001) led to significant upregulation of *α-Klotho* and effectively returned its expression back to baseline levels (Table S2). No difference was observed between the two colchicine-based regimens in terms of thoracic aortic *α-Klotho* expression (Figure 2).

#### 3.2.2. *α-Klotho* in Abdominal Aortic Specimens

*α-Klotho* expression was similar in the abdominal aortas of the four study groups (Table S3).

### 3.3. NANOG Expression

#### 3.3.1. *NANOG* in Thoracic Aortic Specimens

There was a trend towards thoracic *NANOG* upregulation in group A compared to controls. That said, the threshold for statistical significance was not reached. *NANOG* was significantly reduced compared to group A only in the colchicine/NAC subgroup (MD: 4.33, 95% CI: 0.37 to 8.29, *p* = 0.04), (Table S4, Figure S2).

#### 3.3.2. *NANOG* in Abdominal Aortic Specimens

*NANOG* expression was similar in the abdominal aortas of the four treatment arms (Table S5).

### 3.4. NOTCH1 Expression

#### 3.4.1. *NOTCH1* in Thoracic Aortic Specimens

**Figure 3:**
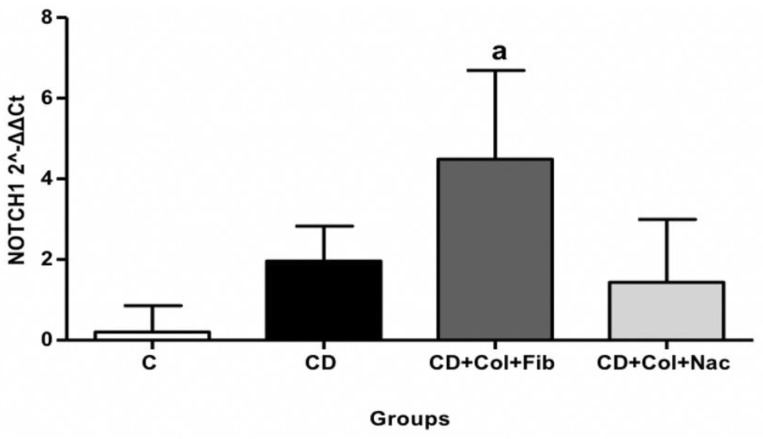
Comparison of *NOTCH1* expression in rabbit thoracic aortas. C: control, CD: cholesterol diet (group A), Col: colchicine, Fib: fibrate, NAC: N-acetylcysteine. a: C vs. CD+Col+Fib.

A hypercholesterolemic diet alone did not significantly affect *NOTCH1* expression patterns in rabbit thoracic aortic specimens. Compared to controls, animals that underwent treatment with colchicine plus fenofibrate while being fed a hyperlipidemic diet exhibited significant *NOTCH1* upregulation in their thoracic aortas (MD: −4.29, 95% CI: −8.09 to −0.48, *p* = 0.03), (Table S6). Interestingly, the combination of colchicine with NAC did not result in a statistically notable increase in thoracic *NOTCH1* expression (Figure 3).

#### 3.4.2. NOTCH1 in Abdominal Aortic Specimens

*NOTCH1* expression was not significantly different in the abdominal aortas of the four animal groups (Table S7).

### 3.5. HIF1a Expression

#### 3.5.1. *HIF1a* in Thoracic Aortic Specimens

*HIF1a* expression was similar in the thoracic aortas of the four study groups (Table S8).

#### 3.5.2. *HIF1a* in Abdominal Aortic Specimens

**Figure 4:**
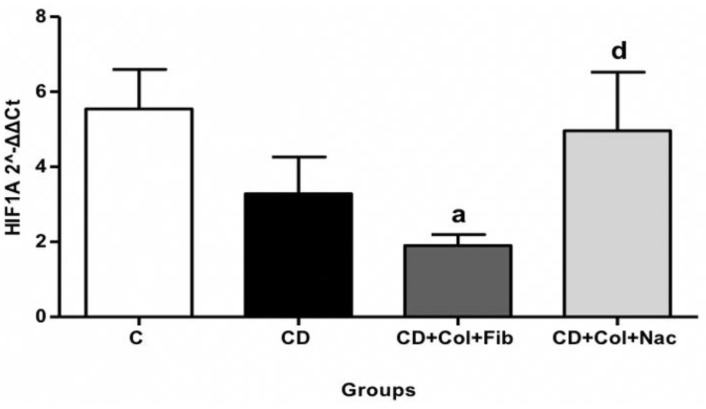
Comparison of *HIF1a* expression in rabbit abdominal aortas. C: control, CD: cholesterol diet (group A), Col: colchicine, Fib: fibrate, NAC: N-acetylcysteine. a = C vs. CD+ Col+Fib; d = C vs. CD+Col+NAC.

*HIF1a* expression in the abdominal aortas of group B animals was significantly reduced compared to the controls (MD: 3.64, 95% CI: 0.83 to 6.44, *p* = 0.03), (Table S9). No difference was observed in terms of abdominal aortic *HIF1a* when comparing group A to groups B and C (Figure 4).

### 3.6. HOXA5 Expression

#### 3.6.1. *HOXA5* in Thoracic Aortic Specimens

*HOXA5* expression was similar in the thoracic aortas of the four study groups (Table S10).

#### 3.6.2. *HOXA5* in Abdominal Aortic Specimens

**Figure 5:**
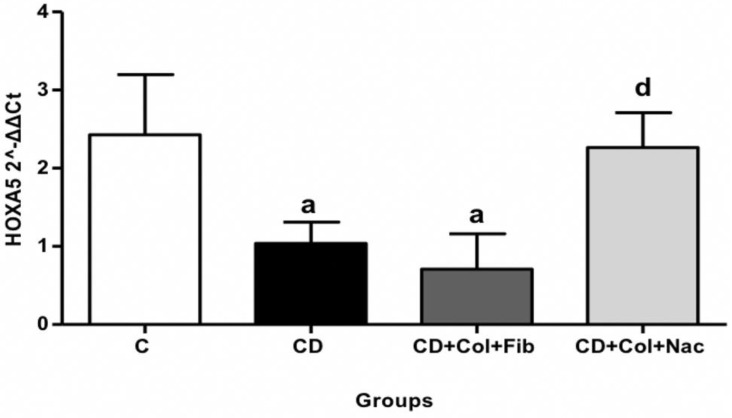
Comparison of *HOXA5* expression in rabbit abdominal aortas. C: control, CD: cholesterol diet (group A), Col: colchicine, Fib: fibrate, NAC: N-acetylcysteine. Footnote: Letters mark statistical significance between groups as follows: a: C vs. CD, CD+Col+Fib; d: C vs. CD+Col+Nac.

*HOXA5* expression was reduced in group A compared to controls (MD: 1.39, 95% CI: 0.03 to 2.74, *p* = 0.02). *HOXA5* remained downregulated in the setting of colchicine/fenofibrate treatment (MD: 1.71, 95% CI: 0.36 to 3.06, *p* = 0.03), (Table S11). Interestingly, animals that received colchicine with NAC exhibited upregulation of *HOXA5* in their abdominal aortas back to the levels of controls (Figure 5).

### 3.7. BMP4, SOX2, and OCT4 Expression

No statistically significant differences were observed in terms of *BMP4*, *SOX2*, and *OCT4* expression in thoracic and abdominal aortic specimens (Tables S12–S17).

### 3.8. Summary of Findings

A synopsis of thoracic aortic findings is provided in Table 1, Tables S18 and S19. Lastly, an overview of abdominal aortic results can be found in Table 2, Tables S20 and S21.

## 4. Discussion

Low shear stress dysregulates developmental signaling pathways in atheroprone regions. This leads to increased inflammation and vascular permeability. Proof-of-concept research from our group has shown that colchicine regimens can attenuate pro-atherogenic inflammation and curtail *KLF4* upregulation in atherosclerotic thoracic aortas [20]. Building upon these benchmark data, we sought to explore the impact of prolonged hyperlipidemia as well as the effect colchicine-based therapy on a variety of atheroprotective (*Klotho, HOXA5, NOTCH1,* and *OCT4*) and proatherogenic (*HIF1a, SOX2, BMP4,* and *NANOG*) genes.

For seven weeks, group A animals were fed a hyperlipidemic diet alone, group B animals were fed the same diet enriched with colchicine and fenofibrate, while group C animals received hypercholesterolemic diet with the addition of colchicine and NAC. All hyperlipidemic groups developed thoracic and abdominal aortic atheromatosis. Animals receiving colchicine-based therapy experienced significantly less thoracic and abdominal aortic atheromatosis compared to their unmedicated hyperlipidemic counterparts. Combining colchicine with NAC instead of fibrate resulted in stronger atheroprotection both in the thoracic and the abdominal aorta.

*Klotho* expression was significantly reduced in the atherosclerotic thoracic aortas of animals receiving hyperlipidemic diet alone compared to controls. Both colchicine/fenofibrate and colchicine/NAC led to significant upregulation of *α-Klotho* and effectively elevated its expression back to baseline levels. No statistically significant difference was observed between the two colchicine-based regimens in terms of thoracic aortic *α-Klotho* expression.

*Klotho* is crucial in maintaining endothelial integrity. In cultured human umbilical endothelial cells, incubation with Klotho halts monocyte adhesion by suppressing TNF α–induced expression of vascular cell adhesion molecule 1 (VCAM1) and intercellular adhesion molecule-1 (ICAM1). It also attenuates NF-κB activation [14]. Additionally, the intracellular form of the Klotho protein can inhibit the retinoid acid-inducible gene-I-induced expression of IL-6 and IL-8 both in vitro and in vivo [28].

Furthermore, *Klotho* attenuates oxidative stress in endothelial and vascular smooth muscle cells (VSMCs). Transfection of cultured VSMCs with *Klotho* has been shown to reduce Nox2 NADPH oxidase protein expression whilst attenuating angiotensin II-induced superoxide production [29]. Similarly, *Klotho* induces the expression of the antioxidant erythroid 2-related factor 2 and upregulates heme oxygenase and peroxiredoxin-1. These enzymes ultimately enhance glutathione levels in human aortic VSMCs [30].

A growing body of literature has also shown that *α-Klotho* protects against vascular calcification by preventing differentiation of VSMCs to an osteoblast-like phenotype [31]. Animals lacking Klotho have increased expression of type III cotransporters (PiT-1/PiT-2) which mediate phosphate-induced VSMC calcification [31,32,33]. Moreover, *Klotho* deficiency induces the osteogenic transcriptional factor CBFA1/RUNX2 in VSMCs which further promotes vascular calcification. On the other hand, the addition of Klotho to VSMCs in vitro, decreases phosphate uptake by suppressing the activity of type III cotransporters and prevents the phenotypic switch of VSMCs to an osteochondrogenic phenotype [31].

In adults without known risk factors for cardiovascular disease (CVD), low serum *Klotho* has been associated with greater carotid artery intima–media thickness and more severe peripheral artery disease. This suggests that reduced serum *Klotho* may be an early predictor of subclinical atherosclerosis [34]. Additional data have shown that patients with significant CAD not only present with lower serum *Klotho* concentration, but also exhibit reduced Klotho mRNA levels in the coronary wall. Interestingly, *Klotho* reduction appears to independently correlate with the severity of CAD [35]. Our study is the first to document recovery of *Klotho* levels in atherosclerotic aortic tissue by colchicine-based treatment.

It should also be emphasized that part of the atheroprotective effect of colchicine/fenofibrate in thoracic aortas was due to *NOTCH1* upregulation. Importantly, *NOTCH1* safeguards endothelial and junctional integrity [15]. It also hinders the expression of pro-inflammatory adhesion molecules including CXCL2, ICAM-1, and CXCR4, and inhibits several interleukins [36]. On the other hand, downregulation of *NOTCH1* promotes cellular proliferation by inducing the overexpression of cyclins and cyclin-dependent kinases. Reduced levels of *NOTCH1* also lead to between-cell instability due to the upregulation of genes involved in intracellular calcium homeostasis, such as CAMK2B and Apelin [37]. Connexin-37 and aconitase levels also increase in the absence of *NOTCH1*—which, in turn, leads to defective gap junctions [38]. Ultimately, intercellular gaps favor the accumulation of fibrin and, thus, enable the attachment of macrophages to vascular endothelium.

The downregulation of *NANOG* also contributed to the atheroprotective effect of colchicine/NAC on thoracic aortas. A growing body of literature has attributed proatherogenic properties to *NANOG*. First, it promotes osteopontin upregulation and VSMC phenotypic switch. Overexpression of *NANOG* also enhances the proliferation, migration, and anti-apoptosis capabilities of vascular SMCs [16]. Lastly, *NANOG* upregulation results in the loss of VE-cadherin from adherens junctions [39].

In the abdominal aorta, hypercholesterolemic diet led to significant downregulation of the atheroprotective *HOXA5* gene. Reduced *HOXA5* expression has been implicated in intimal hyperplasia and derailed angiogenesis [17]. *HOXA5* downregulation also promotes proatherogenic gene expression, extracellular matrix modification, and integrin alterations [40]. Moreover, in the setting of *HOXA5* deficiency, macrophage cells and VSMCs switch towards the proinflammatory M1 phenotype [40].

The combination of colchicine plus NAC reversed *HOXA5* levels back to baseline whereas the coadministration of colchicine with fenofibrate failed to do so. Of note, *HOXA5* upregulation promotes the stabilization of adherens junctions by increasing the retention of beta-catenin. This process diminishes vascular permeability [41]. *HOXA5* also maintains endothelial integrity by regulating key inflammatory mediators, such as thrombospondin-2, vascular endothelial growth factor receptor-2, ephrin-A1, HIF1a, and prostaglandin-endoperoxide synthase-2 [42].

In abdominal aortas, colchicine/fenofibrate downregulated the proatherogenic *HIF1a* gene compared to baseline. Indeed, low hemodynamic shear stress induces *HIF1a* expression in response to localized endothelial hypoxia. *HIF1a* levels are also driven by (1) NF-κB-mediated overproduction and (2) Cezanne-mediated de-ubiquitination which salvages *HIF1a* from proteasomal degradation [43]. *HIF1α* drives atherogenesis in many ways. First, it promotes intraplaque angiogenesis. Second, HIF1α triggers the production of adhesion molecules such as CXCL1, ICAM-1, and VCAM-1 [44,45]. Third, it induces the expression of several enzymes involved in the metabolism of glucose (i.e., phosphofructo-2-kinase/fructose-2,6-biphosphatase 3, hexokinase 2, enolase 2, and glucose transporters 1 and 3) [18]. Fourth, *HIF1α*-induced upregulation of *KLF4* promotes migration of VSMCs, thereby increasing the size of atherosclerotic lesions [46]. HIF1α also induces the phenotypic switch of macrophages and monocytes towards the M1 pro-inflammatory phenotype [47]. The inhibition of the aforementioned processes via colchicine/fenofibrate accounted for at least part of this regimen’s atheroprotective effect on rabbit abdominal aortas.

Last, but not least, no statistically significant differences were noted in terms of the osteogenic *BMP4*, *SOX2,* and *OCT4* genes in thoracic and abdominal aortic specimens [48,49]. This is not surprising considering that no calcified atherosclerotic lesions developed in any of the groups during this seven-week experiment. Indeed, the lack of variation in osteogenic gene expression may be an implication of the relatively short study period rather than an actual effect of colchicine-based treatment.

The present study is subject to several important limitations. First, study animals were unequally assigned to four treatment groups (albeit randomly). The control and group A each comprised six animals, while groups B and C included eight animals. In compliance with the 3R rules (replacement, reduction, and refinement), we sought to minimize the number of animals that had to be utilized [50]. Second, we did not include a colchicine-only group on account of prior research suggesting that colchicine alone may not be enough to halt de novo atherogenesis in hyperlipidemic rabbits [22]. Third, increased *NOTCH1* in macrophages induces M1 and pro inflammatory phenotype. That said, considering the end result of atheroprotection in thoracic aortas, *NOTCH1* is likely upregulated in endothelial cells rather than macrophages. Nevertheless, this premise cannot be confirmed based on qPCR of whole cell lysate. Although, *BMP4* and *SOX2* may also exert opposing effects on different cell types, no statistically significant net associations were observed in our experiment. Fourth, our study did not look at protein production patterns and was not designed to investigate the exact mechanisms through which stem-cell genes moderate atherogenesis. Lastly, cellular staining using stem-cell markers was not performed due to scarcity of resources.

Moving forward, our lab aims to investigate the biomechanics of aortic atheromatosis; indeed, the thoracic and abdominal aorta differ in terms of length, diameter, curvatures, and branch network. These features affect intrinsic hemodynamics and shear stress forces [51,52], and likely account for the variations in atherosclerotic burden and gene dysregulation that were noted in our experiment. We will also be exploring cell-level differences in stem-cell gene expression patterns. Last, but not least, we plan to expand our research in human subjects. Our goal is to explore the correlation between the aortic expression of stem-cell genes and corresponding serum levels in atheromatosis (since these are more convenient to track clinically).

## 5. Conclusions

The expression pattern of aortic stem-cell genes was spatially influenced by Western-type diet and could be modified using colchicine regimens. Hyperlipidemic diet drove de novo thoracic and abdominal aortic atherogenesis by downregulating *α-Klotho* and *HOXA5*, respectively. Both colchicine regimens halted thoracic aortic atheromatosis by upregulating *α-Klotho*. In the thoracic aorta, combining colchicine with fenofibrate also increased *NOTCH1*, while the addition of NAC reduced *NANOG*. In the abdominal aorta, combining colchicine with fenofibrate reduced *HIF1a*, whereas the addition of NAC upregulated *HOXA5*.

## Figures and Tables

**Table 1:** Comparing hyperlipidemic animals to controls in terms of thoracic aortic gene expression.

*Gene*	*CD*	*CD+Col+Fib*	*CD+Col+NAC*
*BMP4*	ns	ns	ns
*SOX2*	ns	ns	ns
*OCT4*	ns	ns	ns
*NANOG*	ns	ns	ns
*NOTCH1*	ns	↑	ns
*HIF1a*	ns	ns	ns
*HOXA5*	ns	ns	ns
*α-Klotho*	↓	ns	ns

*BMP4*: bone morphogenetic protein 4; *SOX2*: Sry-related HMG box 2; *OCT4*: Octamer-binding transcription factor 4; *HIF1α*—hypoxia- inducible factor 1a; C: control, CD: cholesterol diet (group A), Col: colchicine, Fib: fibrate, NAC: N-acetylcysteine; ns: not significant; ↑: gene upregulation; ↓: gene downregulation.

**Table 2:** Comparing hyperlipidemic animals to controls in terms of abdominal aortic gene expression.

*Gene*	*CD*	*CD*+*Col*+*Fib*	*CD*+*Col*+*NAC*
*BMP4*	ns	ns	ns
*SOX2*	ns	ns	ns
*OCT4*	ns	ns	ns
*NANOG*	ns	ns	ns
*NOTCH1*	ns	ns	ns
*HIF1a*	ns	↓	ns
*HOXA5*	↓	↓	ns
*α-Klotho*	ns	ns	ns

*BMP4*: bone morphogenetic protein 4; *SOX2*: Sry-related HMG box 2; *OCT4*: Octamer-binding transcription factor 4; *HIF1α*—hypoxia- inducible factor 1a; C: control, CD: cholesterol diet (group A), Col: colchicine, Fib: fibrate, NAC: N-acetylcysteine; ns: not significant. ↓: gene downregulation.

## Data Availability

All relevant data are presented herein.

## Supplementary Materials

The following supporting information can be downloaded at: https://www.mdpi.com/article/10.3390/jcm11216465/s1, Figure S1: Oil Red O staining of thoracic and abdominal aortic samples per study group (x10 magnification; Figure S2: Comparison of NANOG expression in rabbit thoracic aortas; Table S1: Primer sequences; Table S2: Comparison of α-Klotho expression in rabbit thoracic aortas; Table S3: Comparison of α-Klotho expression in rabbit abdominal aortas; Table S4: Comparison of NANOG expression in rabbit thoracic aortas; Table S5: Comparison of NANOG expression in rabbit abdominal aortas; Table S6: Comparison of NOTCH1 expression in rabbit thoracic aortas; Table S7: Comparison of NOTCH1 expression in rabbit abdominal aortas; Table S8: Comparison of HIF1a expression in rabbit thoracic aortas; Table S9: Comparison of HIF1a expression in rabbit abdominal aortas; Table S10: Comparison of HOXA5 expression in rabbit thoracic aortas; Table S11: Comparison of HOXA5 expression in rabbit abdominal aortas; Table S12: Comparison of BMP4 expression in rabbit thoracic aortas; Table S13: Comparison of BMP4 expression in rabbit abdominal aortas; Table S14: Comparison of SOX2 expression in rabbit thoracic aortas; Table S15: Comparison of SOX2 expression in rabbit abdominal aortas; Table S16: Comparison of OCT4 expression in rabbit thoracic aortas; Table S17: Comparison of OCT4 expression in rabbit abdominal aortas; Table S18: Comparing hyperlipidemic animals receiving colchicine-based regimens to unmedicated hyperlipidemic animals in terms of thoracic aortic gene expression; Table S19: Comparing hyperlipidemic animals receiving colchicine/fenofibrate to colchicine/NAC in terms of thoracic aortic gene expression; Table S20: Comparing hyperlipidemic animals receiving colchicine-based regimens to unmedicated hyperlipidemic animals in terms of abdominal aortic gene expression; Table S21: Comparing hyperlipidemic animals receiving colchicine/fenofibrate to colchicine/NAC in terms of abdominal aortic gene expression.
