# Supplementary material for: Mechanosensitive Stem-Cell Genes and Klotho in Atherosclerotic Aortas: Regulating Spatially Deranged Expression Patterns Using Colchicine Regimens
Source: J Clin Med. 2022 Oct 31;11(21):6465. doi: 10.3390/jcm11216465 (PMC9656280; doi:10.3390/jcm11216465)
Supplement: Supplementary file 1 [file jcm-11-06465-s001.zip › jcm-1928187-Supplementary.pdf]

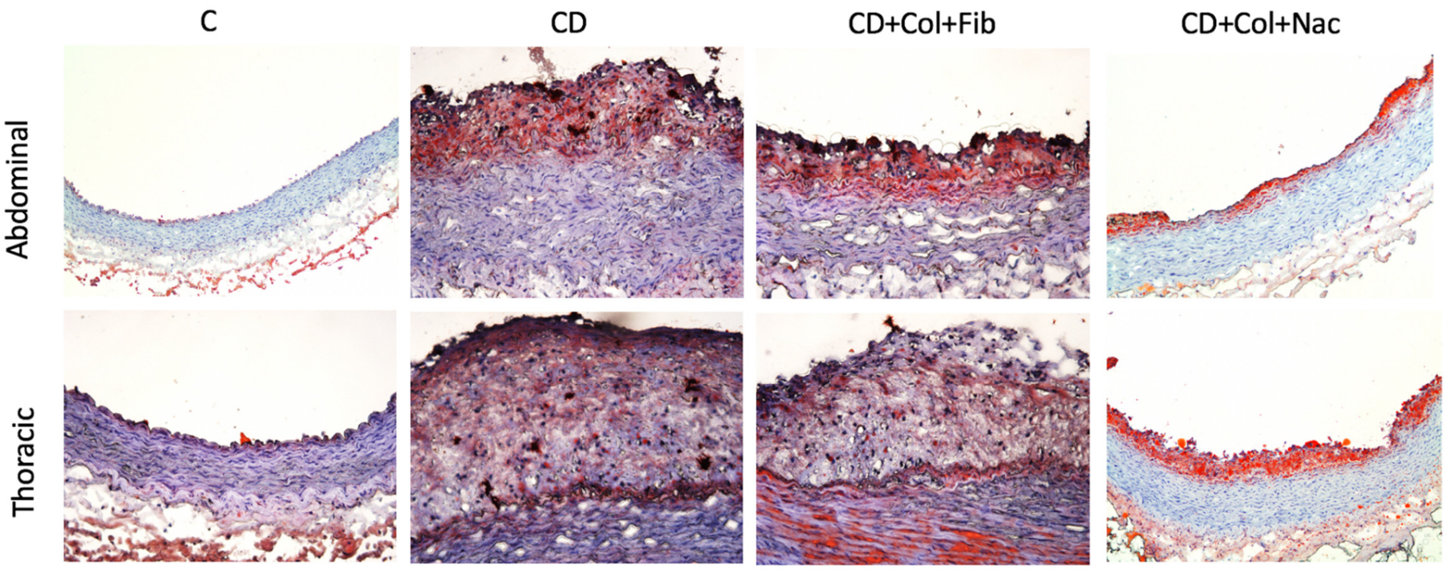

Figure S1. Oil Red O staining of thoracic and abdominal aortic samples per study group (x10 magnification)

C: control, CD: cholesterol diet (group A), Col: colchicine, Fib: fibrate, NAC: N-acetylcysteine

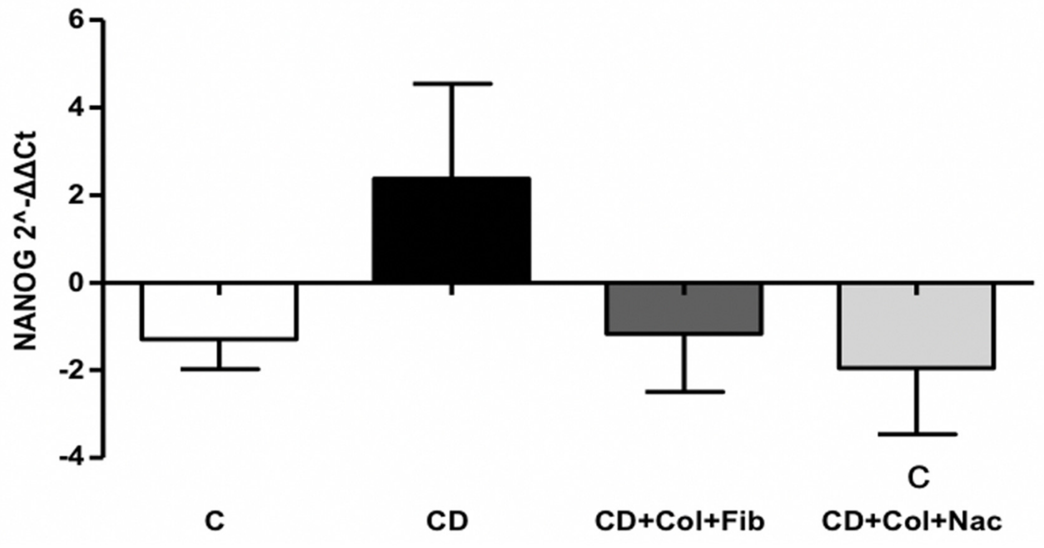

Figure S2. Comparison of *NANOG* expression in rabbit thoracic aortas

C: control, CD: cholesterol diet (group A), Col: colchicine, Fib: fibrate, NAC: N-acetylcysteine

Table S1. Primer sequences

BMP4

	**Sequence (5'->3')**	**Template strand**
**Forward primer**	GGGCTTCCACCGGATAAACA	Plus
**Reverse primer**	CCTGATGGGTCCGTGTATGG	Minus
**Product length**	227	

SOX2

	**Sequence (5'->3')**	**Template strand**
**Forward primer**	CTACAGCATGATGCAGGACCA	Plus
**Reverse primer**	TGGGAGAGCCGTTCATGTAG	Minus
**Product length**	155	

OCT4

	**Sequence (5'->3')**	**Template strand**
**Forward primer**	GCTCCCCCATGCATTCAAAC	Plus
**Reverse primer**	ACTGAATCCCCAAAGCCCTG	Minus
**Product length**	117	

NANOG

	Sequence (5'->3')	Template strand
**Forward primer**	TACCCAGGCTTCTACCCTGC	Plus
**Reverse primer**	GGTTACTCCACGACTGGCTG	Minus
**Product length**	136	

NOTCH1

	Sequence (5'->3')	Template strand
**Forward primer**	GTTCGAGGACCAGATGGCTT	Plus
**Reverse primer**	GATGTTGGCATCTGCACTGG	Minus
**Product length**	243	

HIF1a

	Sequence (5'->3')	Template strand
**Forward primer**	TTTTGGCAGCAACGACACAG	Plus
**Reverse primer**	GTGCAGGGTCAGCACTACTT	Minus
**Product length**	173	

HOXA5

	Sequence (5'->3')	Template strand

**Forward primer**	CATGCTCTTTGCCTCTCCGA	Plus
**Reverse primer**	TTAGGGCAACGAGAACAGGG	Minus
**Product length**	248	

a-Klotho

	**Sequence (5'->3')**	**Template strand**
**Forward primer**	CAGCGACGGCTACAACAATG	Plus
**Reverse primer**	AGCAGAGTTCGGCGTAATCC	Minus
**Product length**	296	

GAPDH

	**Sequence (5'->3')**	**Template strand**
**Forward primer**	AGGGCTGCTTTTAACTCTGGT	Plus
**Reverse primer**	CCCCACTTGATTTTGGAGGGA	Minus

BMP4: bone morphogenetic protein 4; SOX2: Sry-related HMG box 2; OCT4: Octamer-binding transcription factor 4; HIF1α - hypoxia- inducible factor 1a; GAPDH - glyceraldehyde 3-phosphate dehydrogenase

Table S2. Comparison of *α-Klotho* expression in rabbit thoracic aortas

**ANOVA with Tukey's correction**	**Mean Difference**	**95% CI of difference**	**p-value**
C vs CD	*8.79*	*1.82 to 15.76*	***<0.001***
C vs CD+Col+Fib	-1.24	-8.21 to 5.72	>0.05
C vs CD+Col+NAC	1.31	-5.04 to 7.67	>0.05
CD vs CD+Col+Fib	-10.04	-17.00 to -3.07	***<0.001***
CD vs CD+Col+NAC	-7.47	-13.83 to -1.11	***<0.001***
CD+Col+Fib vs CD+Col+NAC	2.56	-3.79 to 8.92	>0.05

ANOVA: analysis of variance; CI: confidence interval; C: control, CD: cholesterol diet (group A), Col: colchicine, Fib: fibrate, NAC: N-acetylcysteine

Table S3. Comparison of *α-Klotho* expression in rabbit abdominal aortas

ANOVA with Tukey's correction	*Mean Difference*	*95% CI of difference*	*p-value*
C vs CD	-1.33	-6.57 to 3.90	>0.05
C vs CD+Col+Fib	-1.87	-6.65 to 2.90	>0.05
C vs CD+Col+NAC	-0.34	-5.12 to 4.44	>0.05
CD vs CD+Col+Fib	-0.54	-5.32 to 4.23	>0.05
CD vs CD+Col+NAC	0.99	-3.78 to 5.77	>0.05
CD+Col+Fib vs CD+Col+NAC	1.53	-2.74 to 5.81	>0.05

ANOVA: analysis of variance; CI: confidence interval; C: control, CD: cholesterol diet (group A), Col: colchicine, Fib: fibrate, NAC: N-acetylcysteine

Table S4. Comparison of *NANOG* expression in rabbit thoracic aortas

ANOVA with Tukey's correction	***Mean Difference***	***95% CI of difference***	***p-value***
C vs CD	-3.67	-7.64 to 0.28	0.06
C vs CD+Col+Fib	-0.12	-4.08 to 3.84	>0.05
C vs CD+Col+NAC	0.65	-3.30 to 4.62	>0.05
CD vs CD+Col+Fib	3.55	-0.40 to 7.51	0.06
CD vs CD+Col+NAC	4.33	0.37 to 8.29	***0.04***
CD+Col+Fib vs CD+Col+NAC	0.78	-3.18 to 4.74	>0.05

ANOVA: analysis of variance; CI: confidence interval; C: control, CD: cholesterol diet (group A), Col: colchicine, Fib: fibrate, NAC: N-acetylcysteine

Table S5. Comparison of *NANOG* expression in rabbit abdominal aortas

ANOVA with Tukey's correction	***Mean Difference***	***95% CI of difference***	***p-value***
C vs CD	1.62	-2.23 to 4.61	>0.05
C vs CD+Col+Fib	0.09	-3.01 to 3.12	>0.05
C vs CD+Col+NAC	2.41	-1.48 to 4.64	>0.05
CD vs CD+Col+Fib	1.54	-4.55 to 2.29	>0.05
CD vs CD+Col+NAC	0.53	-3.03 to 3.82	>0.05
CD+Col+Fib vs CD+Col+NAC	2.32	-1.54 to 4.58	>0.05

ANOVA: analysis of variance; CI: confidence interval; C: control, CD: cholesterol diet (group A), Col: colchicine, Fib: fibrate, NAC: N-acetylcysteine

Table S6. Comparison of *NOTCH1* expression in rabbit thoracic aortas

ANOVA with Tukey's correction	***Mean Difference***	***95% CI of difference***	***p-value***
C vs CD	-1.76	-5.56 to 2.04	>0.05
C vs CD+Col+Fib	-4.29	-8.09 to -0.48	***0.03***
C vs CD+Col+NAC	-1.24	-5.04 to 2.56	>0.05
CD vs CD+Col+Fib	-2.53	-6.33 to 1.27	>0.05
CD vs CD+Col+NAC	0.52	-3.28 to 4.32	>0.05
CD+Col+Fib vs CD+Col+NAC	3.05	-0.75 to 6.85	>0.05

ANOVA: analysis of variance; CI: confidence interval; C: control, CD: cholesterol diet (group A), Col: colchicine, Fib: fibrate, NAC: N-acetylcysteine

Table S7. Comparison of *NOTCH1* expression in rabbit abdominal aortas

ANOVA with Tukey's correction	***Mean Difference***	***95% CI of difference***	***p-value***
C vs CD	-0.28	-6.48 to 5.92	>0.05
C vs CD+Col+Fib	-0.23	-6.43 to 5.97	>0.05
C vs CD+Col+NAC	1.07	-5.12 to 7.28	>0.05
CD vs CD+Col+Fib	0.05	-5.50 to 5.60	>0.05
CD vs CD+Col+NAC	1.36	-4.19 to 6.91	>0.05
CD+Col+Fib vs CD+Col+NAC	1.31	-4.24 to 6.86	>0.05

ANOVA: analysis of variance; CI: confidence interval; C: control, CD: cholesterol diet (group A), Col: colchicine, Fib: fibrate, NAC: N-acetylcysteine

Table S8. Comparison of *HIF1a* expression in rabbit thoracic aortas

ANOVA with Tukey's correction	***Mean Difference***	***95% CI of difference***	***p-value***
C vs CD	2.52	-4.17 to 9.21	>0.05
C vs CD+Col+Fib	-1.38	-8.08 to 5.31	>0.05
C vs CD+Col+NAC	4.90	-1.79 to 11.60	>0.05
CD vs CD+Col+Fib	-3.90	-10.60 to 2.79	>0.05
CD vs CD+Col+NAC	2.38	-4.31 to 9.07	>0.05
CD+Col+Fib vs CD+Col+NAC	6.28	-0.41 to 12.98	>0.05

ANOVA: analysis of variance; CI: confidence interval; C: control, CD: cholesterol diet (group A), Col: colchicine, Fib: fibrate, NAC: N-acetylcysteine

Table S9. Comparison of *HIF1a* expression in rabbit abdominal aortas

ANOVA with Tukey's correction	***Mean Difference***	***95% CI of difference***	***p-value***
C vs CD	2.26	-0.54 to 5.06	>0.05
C vs CD+Col+Fib	3.64	0.83 to 6.44	***0.03***
C vs CD+Col+NAC	0.58	-2.21 to 3.38	>0.05
CD vs CD+Col+Fib	1.38	-1.42 to 4.18	>0.05
CD vs CD+Col+NAC	-1.67	-4.47 to 1.12	>0.05
CD+Col+Fib vs CD+Col+NAC	-3.05	-5.85 to -0.25	***0.03***

ANOVA: analysis of variance; CI: confidence interval; C: control, CD: cholesterol diet (group A), Col: colchicine, Fib: fibrate, NAC: N-acetylcysteine

Table S10. Comparison of *HOXA5* expression in rabbit thoracic aortas

ANOVA with Tukey's correction	*Mean Difference*	*95% CI of difference*	*p-value*
C vs CD	0.70	-3.49 to 4.90	>0.05
C vs CD+Col+Fib	0.36	-3.38 to 4.11	>0.05
C vs CD+Col+NAC	1.62	-2.13 to 5.37	>0.05
CD vs CD+Col+Fib	-0.33	-4.53 to 3.85	>0.05
CD vs CD+Col+NAC	0.91	-3.28 to 5.11	>0.05
CD+Col+Fib vs CD+Col+NAC	1.25	-2.49 to 5.01	>0.05

ANOVA: analysis of variance; CI: confidence interval; C: control, CD: cholesterol diet (group A), Col: colchicine, Fib: fibrate, NAC: N-acetylcysteine

Table S11. Comparison of *HOXA5* expression in rabbit abdominal aortas

ANOVA with Tukey's correction	*Mean Difference*	*95% CI of difference*	*p-value*
C vs CD	1.39	0.03 to 2.74	***0.03***
C vs CD+Col+Fib	1.71	0.36 to 3.06	***0.02***
C vs CD+Col+NAC	0.16	-1.19 to 1.51	>0.05
CD vs CD+Col+Fib	0.32	-1.02 to 1.67	>0.05
CD vs CD+Col+NAC	-1.23	-2.58 to 0.12	>0.05
CD+Col+Fib vs CD+Col+NAC	-1.55	-2.90 to -0.20	***0.03***

ANOVA: analysis of variance; CI: confidence interval; C: control, CD: cholesterol diet (group A), Col: colchicine, Fib: fibrate, NAC: N-acetylcysteine

Table S12. Comparison of *BMP4* expression in rabbit thoracic aortas

ANOVA with Tukey's correction	*Mean Difference*	*95% CI of difference*	*p-value*
C vs CD	-2.16	-5.83 to 1.50	>0.05
C vs CD+Col+Fib	-0.79	-4.46 to 2.87	>0.05
C vs CD+Col+NAC	-1.41	-5.08 to 2.26	>0.05
CD vs CD+Col+Fib	1.37	-1.91 to 4.65	>0.05
CD vs CD+Col+NAC	0.75	-2.52 to 4.04	>0.05
CD+Col+Fib vs CD+Col+NAC	-0.61	-3.90 to 2.66	>0.05

ANOVA: analysis of variance; CI: confidence interval; C: control, CD: cholesterol diet (group A), Col: colchicine, Fib: fibrate, NAC: N-acetylcysteine

Table S13. Comparison of *BMP4* expression in rabbit abdominal aortas

ANOVA with Tukey's correction	*Mean Difference*	*95% CI of difference*	*p-value*
C vs CD	-0.28	-2.06 to 1.50	>0.05
C vs CD+Col+Fib	-1.44	-3.22 to 0.34	>0.05
C vs CD+Col+NAC	-0.31	-2.09 to 1.47	>0.05
CD vs CD+Col+Fib	-1.16	-2.94 to 0.62	>0.05
CD vs CD+Col+NAC	-0.03	-1.81 to 1.75	>0.05
CD+Col+Fib vs CD+Col+NAC	1.13	-0.65 to 2.91	>0.05

ANOVA: analysis of variance; CI: confidence interval; C: control, CD: cholesterol diet (group A), Col: colchicine, Fib: fibrate, NAC: N-acetylcysteine

Table S14. Comparison of *SOX2* expression in rabbit thoracic aortas

ANOVA with Tukey's correction	***Mean Difference***	***95% CI of difference***	***p-value***
C vs CD	2.65	-2.43 to 7.73	>0.05
C vs CD+Col+Fib	-1.05	-6.13 to 4.03	>0.05
C vs CD+Col+NAC	-1.52	-6.60 to 3.55	>0.05
CD vs CD+Col+Fib	-3.70	-8.78 to 1.38	>0.05
CD vs CD+Col+NAC	-4.17	-9.25 to 0.90	>0.05
CD+Col+Fib vs CD+Col+NAC	-0.47	-5.55 to 4.60	>0.05

ANOVA: analysis of variance; CI: confidence interval; C: control, CD: cholesterol diet (group A), Col: colchicine, Fib: fibrate, NAC: N-acetylcysteine

Table S15. Comparison of *SOX2* expression in rabbit abdominal aortas

ANOVA with Tukey's correction	***Mean Difference***	***95% CI of difference***	***p-value***
C vs CD	2.10	-5.97 to 2.26	>0.05
C vs CD+Col+Fib	2.37	-5.55 to 1.81	>0.05
C vs CD+Col+NAC	1.19	-4.62 to 2.74	>0.05
CD vs CD+Col+Fib	0.01	-4.13 to 4.10	>0.05
CD vs CD+Col+NAC	1.03	-3.20 to 5.03	>0.05
CD+Col+Fib vs CD+Col+NAC	1.17	-2.76 to 4.60	>0.05

ANOVA: analysis of variance; CI: confidence interval; C: control, CD: cholesterol diet (group A), Col: colchicine, Fib: fibrate, NAC: N-acetylcysteine

Table S16. Comparison of *OCT4* expression in rabbit thoracic aortas

ANOVA with Tukey's correction	*Mean Difference*	*95% CI of difference*	*p-value*
C vs CD	-2.87	-6.34 to 0.60	>0.05
C vs CD+Col+Fib	-1.03	-4.13 to 2.07	>0.05
C vs CD+Col+NAC	-2.38	-5.48 to 0.72	>0.05
CD vs CD+Col+Fib	1.84	-1.63 to 5.31	>0.05
CD vs CD+Col+NAC	0.48	-2.98 to 3.95	>0.05
CD+Col+Fib vs CD+Col+NAC	-1.35	-4.45 to 1.75	>0.05

ANOVA: analysis of variance; CI: confidence interval; C: control, CD: cholesterol diet (group A), Col: colchicine, Fib: fibrate, NAC: N-acetylcysteine

Table S17. Comparison of *OCT4* expression in rabbit abdominal aortas

ANOVA with Tukey's correction	*Mean Difference*	*95% CI of difference*	*p-value*
C vs CD	0.73	-3.44 to 4.90	>0.05
C vs CD+Col+Fib	0.24	-3.93 to 4.41	>0.05
C vs CD+Col+NAC	1.06	-3.11 to 5.23	>0.05
CD vs CD+Col+Fib	-0.49	-4.66 to 3.68	>0.05
CD vs CD+Col+NAC	0.32	-3.84 to 4.50	>0.05
CD+Col+Fib vs CD+Col+NAC	0.82	-3.35 to 4.99	>0.05

ANOVA: analysis of variance; CI: confidence interval; C: control, CD: cholesterol diet (group A), Col: colchicine, Fib: fibrate, NAC: N-acetylcysteine

Table S18. Comparing hyperlipidemic animals receiving colchicine-based regimens to unmedicated hyperlipidemic animals in terms of thoracic aortic gene expression

Gene	***CD+Col+Fib***	***CD+Col+NAC***
BMP4	ns	ns
SOX2	ns	ns
OCT4	ns	ns
NANOG	ns	**↓, *p=0.04***
NOTCH1	ns	ns
HIF1a	ns	ns
HOXA5	ns	ns
α-Klotho	**↑, *p<0.001***	**↑, *p<0.001***

BMP4: bone morphogenetic protein 4; SOX2: Sry-related HMG box 2; OCT4: Octamer-binding transcription factor 4; HIF1α - hypoxia- inducible factor 1a; C: control, CD: cholesterol diet (group A), Col: colchicine, Fib: fibrate, NAC: N-acetylcysteine; ns: not significant

Table S19. Comparing hyperlipidemic animals receiving colchicine/fenofibrate to colchicine/NAC in terms of thoracic aortic gene expression

Gene	***Colchicine-based regimens***
BMP4	ns
SOX2	ns
OCT4	ns
NANOG	ns
NOTCH1	ns
HIF1a	ns
HOXA5	ns
α-Klotho	ns

BMP4: bone morphogenetic protein 4; SOX2: Sry-related HMG box 2; OCT4: Octamer-binding transcription factor 4; HIF1α - hypoxia- inducible factor 1a

Table S20. Comparing hyperlipidemic animals receiving colchicine-based regimens to unmedicated hyperlipidemic animals in terms of abdominal aortic gene expression

Gene	***CD+Col+Fib***	***CD+Col+NAC***
BMP4	ns	ns
SOX2	ns	ns
OCT4	ns	ns
NANOG	ns	ns
NOTCH1	ns	ns
HIF1a	ns	ns
HOXA5	ns	ns
α-Klotho	ns	ns

BMP4: bone morphogenetic protein 4; SOX2: Sry-related HMG box 2; OCT4: Octamer-binding transcription factor 4; HIF1α - hypoxia- inducible factor 1a; C: control, CD: cholesterol diet (group A), Col: colchicine, Fib: fibrate, NAC: N-acetylcysteine; ns: not significant

Table S21. Comparing hyperlipidemic animals receiving colchicine/fenofibrate to colchicine/NAC in terms of abdominal aortic gene expression

Gene	***Colchicine-based regimens***
BMP4	ns
SOX2	ns
OCT4	ns
NANOG	ns
NOTCH1	ns
HIF1a	ns
HOXA5	**↑NAC, *p=0.03***
α-Klotho	ns

BMP4: bone morphogenetic protein 4; SOX2: Sry-related HMG box 2; OCT4: Octamer-binding transcription factor 4; HIF1α - hypoxia- inducible factor 1a; NAC: N-acetylcysteine
